# Central Crosstalk for Somatic Tinnitus: Abnormal Vergence Eye Movements

**DOI:** 10.1371/journal.pone.0011845

**Published:** 2010-07-28

**Authors:** Qing Yang, Marine Vernet, Christophe Orssaud, Pierre Bonfils, Alain Londero, Zoi Kapoula

**Affiliations:** 1 Group IRIS, CNRS, Service d'Ophtalmologie-ORL-Stomatologie, Hôpital Européen Georges Pompidou, Paris, France; 2 Service d'Ophtalmologie, Hôpital Européen Georges Pompidou, Paris, France; 3 Service d'ORL et de Chirurgie Cervico-Faciale, Hôpital Européen Georges Pompidou, Faculté de médecine Paris-Descartes, Université Paris V et Laboratoire CNRS UMR 7060, Paris, France; Kyushu University, Japan

## Abstract

**Background:**

Frequent oulomotricity problems with orthoptic testing were reported in patients with tinnitus. This study examines with objective recordings vergence eye movements in patients with somatic tinnitus patients with ability to modify their subjective tinnitus percept by various movements, such as jaw, neck, eye movements or skin pressure.

**Methods:**

Vergence eye movements were recorded with the Eyelink II video system in 15 (23–63 years) control adults and 19 (36–62 years) subjects with somatic tinnitus.

**Findings:**

1) Accuracy of divergence but not of convergence was lower in subjects with somatic tinnitus than in control subjects. 2) Vergence duration was longer and peak velocity was lower in subjects with somatic tinnitus than in control subjects. 3) The number of embedded saccades and the amplitude of saccades coinciding with the peak velocity of vergence were higher for tinnitus subjects. Yet, saccades did not increase peak velocity of vergence for tinnitus subjects, but they did so for controls. 4) In contrast, there was no significant difference of vergence latency between these two groups.

**Interpretation:**

The results suggest dysfunction of vergence areas involving cortical-brainstem-cerebellar circuits. We hypothesize that central auditory dysfunction related to tinnitus percept could trigger mild cerebellar-brainstem dysfunction or that tinnitus and vergence dysfunction could both be manifestations of mild cortical-brainstem-cerebellar syndrome reflecting abnormal cross-modality interactions between vergence eye movements and auditory signals.

## Introduction

Subjective tinnitus (ST) is a sound percept without recordable source. Its pathophysiology remains unclear but it is supposed to result from hyperactivity and neuroplastic reorganization of cortical-subcortical auditory and non-auditory networks [1]. Tinnitus is usually related to an auditory impairment with a good correlation between frequency and laterality of hearing loss and ST percept [2]. But other non-auditory medical conditions like temporomandibular joint and upper spine disorders or whiplash injuries have been associated with subjective tinnitus. Modulation of tinnitus by head, neck or eye movements has been frequently described [3] defining a “somatic tinnitus” subgroup of ST patients. On another hand eye movements abnormality have already been shown in patients with somatic tinnitus, e.g. morphological and gain decrease in smooth pursuit, incorrect optokinetic nystagmus, and disturbance in saccadic eye movements [4], [5], [6]. However, there is still no study on vergence eye movements in patients with ST.

During vergence eye movements, the eyes rotate in opposite direction allowing an adjustment of angle of optic axes according to the depth. Control of these complex eye movements involves the occipital-parietal-frontal cortex [7], [8], [9], the superior colliculus [10], [11], the brainstem [12] and the cerebellum [13], [14], [15]. At the cortical level, an EEG study [7] showed that spatial distribution of EEG activation was more distributed bilaterally for vergence than for saccades. Convergence targets activated a rather extended cortical network in the central and posterior area, while divergence targets activated a more confined posterior area spreading ventrally from the occipital cortex. The cerebellum is also highly involved in vergence control, both online (direct effect [16], [17], [18]) and offline (adaptation effect [19], [20]). For example, the study of Nitta et al. [17], [18] examined simple-spike activity of Purkinje (P) cells in cerebellar dorsal vermis and found majority of the vergence-related P-cells displayed both vergence eye position and velocity sensitivity during the execution of vergence eye movements. Takagi et al. [20] examined vergence eye movements undergoing adaptive recalibration in response to a training stimulus in which the initial disparity is changed just after the vergence movement begins. They found that the dynamics of vergence were changed after adaptation. Similar changes were observed for saccades and the initiation of pursuit eye movements, suggesting common neural mechanisms including cerebellar vermis for adaptive changes in the control of all eye movements [19], [20]. Moreover, patients with cerebellar lesions showed deficits of vergence [13], [21], [22]. Cerebellum is even more important for vergence as not only the dorsal vermis and caudal fastigial nucleus [22] but also the cerebellar flocculus [23] and cerebellar hemisphere [14] are involved in the control of vergence. In addition, neurons in the mesencephalic reticular formation (MRF) were found to be involved in the control of vergence [12], [15], [22]. Therefore, vergence oculomotor system involves highly cortical-subcortical and cerebellar areas. Moreover, it is fragile, subject to aging, fatigue and neurological insults [22], [24], [25]. Lasting vergence problems have been reported in patients with mild brain injuries [26].

In tinnitus patients the hypothetical abnormal connections between auditory and somatosensory centers implying dorsal root ganglia, trigeminal ganglion and brainstem might also involve areas important for various types of eye movements including vergence [27]. Our recent study reported abnormalities of fixation, smooth pursuit and optokinetic nystagmus in 5 cases of such patients [4], particularly deficits for vertical pursuit eye movements and fixation instability for tinnitus patients, which is line with cerebellar dysfunction.

This study examines with objective recordings vergence eye movements in patients presenting ST with ability to modify their ST percept by various movements.

## Materials and Methods

### Ethics statement

The eye movement investigation adhered to the tenets of the Declaration of Helsinki and was approved by the local human experimentation committee, CPP Il de France II (No: 07035), Hospital Necker in Paris. Written consent was obtained from all subjects after the nature of the examination had been explained.

### Patients

All patients attended a tertiary care tinnitus clinic at European Hospital Georges Pompidou in Paris; tinnitus perception being stated as their main medical complaint. They were selected because they showed in common modulation of their tinnitus perception by oro-facial, neck or eye movements or other musculo-skeletal activation (skin or muscle pressure). Epidemiological data are given in Table 1. All patients suffered from tinnitus for at least one year (mean 4.2 years). Mean age was 48.1±12.7 years; 7 females and 12 males. Tinnitus was left sided in 9 patients, right sided in 2 patient and both sides in 8 patients (R>L for 7 and L>R for one patient). Tinnitus was modulated by jaw movements in 14 patients, head movements in 8 patients, skin or muscle pressure in 8 patients, eye movements in 1 patient, global muscular effort in 1. One condition elicited tinnitus modulation in 7 patients, two different conditions in 8 patients, and three in the remaining 3 patients. Tinnitus was considered as idiopathic in 3 patients. Unusual stressful circumstances were present for 8 patients at tinnitus onset. Four patients had an acute otological problem (otitis media (2), otosclerosis surgery and noise induced hearing loss). One had a congenital sensory neural hearing loss. Hearing loss was variable among patients. Hearing was normal in 6 patients (thresholds >15 dB from 250 to 8000 Hz). Various degree of high or middle frequencies SNHL were present in 12 patients and one and unilateral cophosis. None of these patients had significant conductive hearing loss (more than 15 dB of difference on 2 adjacent frequencies). Neurological or muscular impairments were present in 3 patients (acoustic neuroma, meningioma, cervicalgia). Patient 15 with history of surgically treated acoustic neuroma by translabyrinthine approach more than five years before testing experienced dizziness during tinnitus time but had no acute clinical vestibular dysfunction at the time of testing as attested by the absence of vertigo or dizziness or spontaneous nystagmus. Patient T18 had unilateral SNHL (*Sensori- Neural Hearing Loss*) with tinnitus and homolateral meningioma of the posterior fossa discovered by MRI but it is impossible to be affirmative regarding the causal link between these two medical conditions. As mentioned above it should be emphasized that tinnitus is a symptom that can be the consequence of a large variety of diseases, including auditory (i.e. peripheral) and/or neurological (i.e. central) disorders. Thus for all patients clinical information given in Table 1 only indicates the medical conditions that were presented at the onset of tinnitus; however, it is not certain that this was the exclusive cause of tinnitus. None of them had acute clinical vestibular dysfunction at the time of testing as attested by the absence of vertigo or dizziness or spontaneous nystagmus.

**Table 1:** Clinical characteristics in tinnitus patients.

Subjects	Gender	Age (Year)	Aetiology	Side Tinnitus	Pitch	Audiogram	Movement modulation
T1	M	42	Idiopathic	R	High	Normal	**Jaw**
T2	M	43	Idiopathic	L	High	Normal	**Jaw**
T3	M	47	Stress	L	High	High fHZ SNHL	**Jaw, Skin pressure, effort**
T4	F	46	Stress	L	High WN	High fHZ SNHL	**Jaw, Skin pressure**
T5	M	40	Stress	L	High	Normal	**Head, Jaw**
T6	M	61	Stress	L	WN	High fHZ SNHL	**Jaw, Skin pressure**
T7	M	29	Stress	L	High	Normal	**Jaw**
T8	F	58	Stress	R>L	High WN	High fHZ SNHL	**Jaw, Head, Skin pressure**
T9	M	59	Congenital SNHL	R>L	WN	Middle fHZ SNHL	**Head**
T10	M	43	Otitis Media Stress	R	High	Normal	**Skin pressure**
T11	F	41	Stress	R>L	WN	High fHZ SNHL	**Jaw, Skin pressure**
T12	M	51	Noise induced HL	R>L	High	High fHZ SNHL 4	**Jaw, Skin pressure**
T13	F	42	Stress	L	WN	Middle fHZ SNHL	**Jaw, Head**
T14	F	21	Idiopathic	L	High	Normal	**Effort**
T15	M	54	Acoustic neuroma	R>L	High WN	Right cophosis	**Head, Eye**
T16	M	43	SOM, stress	L>R	High WN	High fHZ SNHL	**Head**
T17	F	62	Cervicalgia	L	High	High fHZ SNHL	**Head, Jaw**
T18	F	78	Menigioma	R>L	High	High fHZ SNHL	**Head, Jaw, Skin pressure**
**T19**	**M**	**54**	**Stress**	**R>L**	**High WN**	**High fHZ SNHL**	**Head, Jaw,**

### Visual display

**Figure 1:**
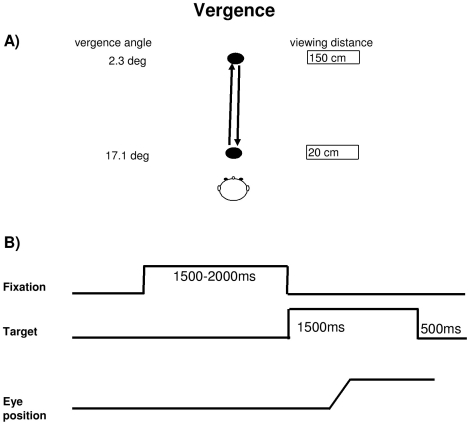
The Vergence eye movement task. (A) Spatial arrangement for vergence: two diodes on an horizontal plane, one at 20 cm (required convergence 17°) and another 150 cm (required vergence 2.3°) from the subject's eyes. (B) Paradigm used for the stimulation: the fixation LED stayed on for a random period between 1.5 and 2 sec; the target LED was kept on for 1.5 sec; a black period of 500 ms was used for break.

The visual display on a horizontal table consisted of circular LEDs (each LED on 2.9 mm, wavelength 636 nm with intensity of luminous 60 mcd) placed at two viewing distance in middle line, one at 20 cm from the subject, and the other at 150 cm. Fixation of the first LED requires vergence angle of 17.1° and fixation of the second LED requires vergence angle of 2.3° (Figure 1A). In a dark room, subject was seated in an adapted chair with a chin and frontal rest. He/she viewed binocularly and faced the visual display of the LEDs. Vertically, all target LEDs were placed at eye level.

### Oculomotor tasks

Each trial started by lighting a fixation LED at the center. The fixation LED stayed on for a random period between 1.5 and 2 sec. The target LED was kept on for 1.5 sec (Figure 1B). Subjects were required to initiate a vergence to the other central target LED as rapidly and accurately as possible. A black period of 500 ms separated trials. Subjects were instructed to use this period for blinks. The total mean length of each trial was about 4 sec. Subject performed one block which contained 30 trials for both divergence (from 20 cm to 150 cm) and convergence (from 150 to 20 cm) interleaved randomly at equal rates.

A calibration sequence was performed at the beginning and at the end of each block; the target made the following predictive sequence for each viewing distance: center, 5° to left, center, 10° to left, center, 5° to right, center, 10° to right, center; the target stayed at each location for 2 sec. From these recordings we extracted calibration factors.

### Eye movement recording

Horizontal eye movements were recorded binocularly with the EyeLink II device. Each channel was sampled at 250 Hz. The system has a spatial resolution of 0.025° in pupil-CR mode and saccade event resolution of 0.05° for microsaccades (see manufacturer specification).

### Data analysis

**Figure 2:**
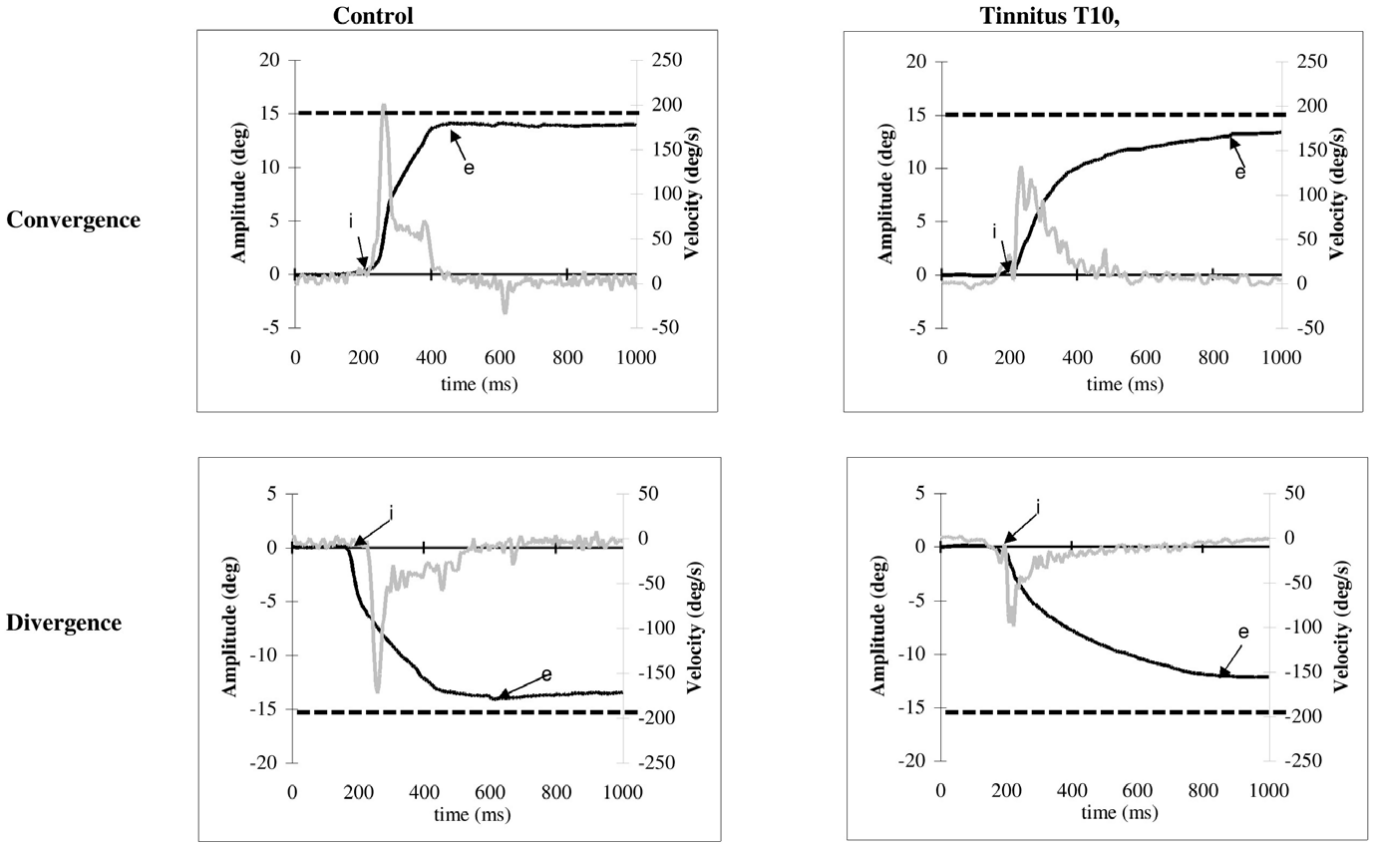
Typical recordings of vergence eye movements. Convergence and divergence with their corresponding velocity traces plotted at different scales are obtained by difference of the position signal between the two eyes (LE-RE); the arrows ‘i’ and ‘p’ indicate the onset and the offset of convergence, respectively; the dashed line indicates the required convergence change.

From the two individual calibrated eyes position signals we derived the disconjugate signal (left eye-right eye). The eye velocity of either conjugate (saccades) or disconjugate (vergence) signal was computed using a symmetrical two-point differentiator after low-pass filtering with a Gaussian FIR filter with a cut-off frequency of 33 Hz. The onset and the offset of the vergence eye movements were defined as the time point when the vergence velocity exceeds or drops 5°/s (Figure 2, point ‘i’ and ‘e’). This criterion is standard [28], [29]. The process was performed automatically by the computer, and the verification was made by visual inspection of the individual eye position and velocity trace.

For both convergence and divergence, we measured the latency, i.e. the time between target onset (0 ms) and vergence onset (marker ‘i’ in Figure 2), accuracy (gain), i.e. ratio of the amplitude of vergence (‘i’ to ‘e’ at position trace, Figure 2) over the amplitude of the target excursion in depth, and the peak velocity (value ‘v’ at the velocity trace, Figure 2). In addition, number of embedded saccades and amplitude of saccades coinciding with the peak velocity of vergence were also analyzed.

Eye movements in the wrong direction, with latency shorter than 80 ms (anticipation) or longer than 800 ms, or contaminated by blinks were rejected. For adults seven percent of trials and for elderly subjects nine percent of trials had to be rejected using these criteria.

A two-way analysis of variance (ANOVA) was performed on individual mean values of each parameter with the between subjects factor-the group (control, tinnitus), and the within subjects factor - vergence (convergence, divergence). Post-hoc comparisons were done with the Least Significant Differences test. The correlation was evaluated with the Spearman test.

## Results

### Latency

**Figure 3:**
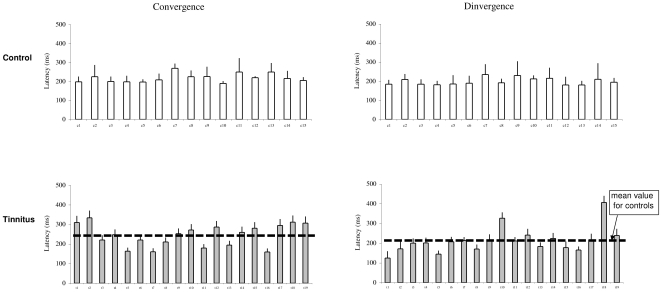
The latency of vergence eye movements. Individual mean latency with standard deviation for convergence and divergence in control and tinnitus subjects.

The individual mean latencies and the standard deviation are shown for convergence and divergence in controls and tinnitus in Figure 3. The two-way ANOVA applied on the latency values shows no effect of group (F_1,32_ = 2.31, p = 0.14), but a significant effect of type of vergence (F_1,32_ = 7.99, p<0.01), i.e. longer latencies for convergence than for divergence. This is the case for both control and tinnitus.

### Gain

**Figure 4:**
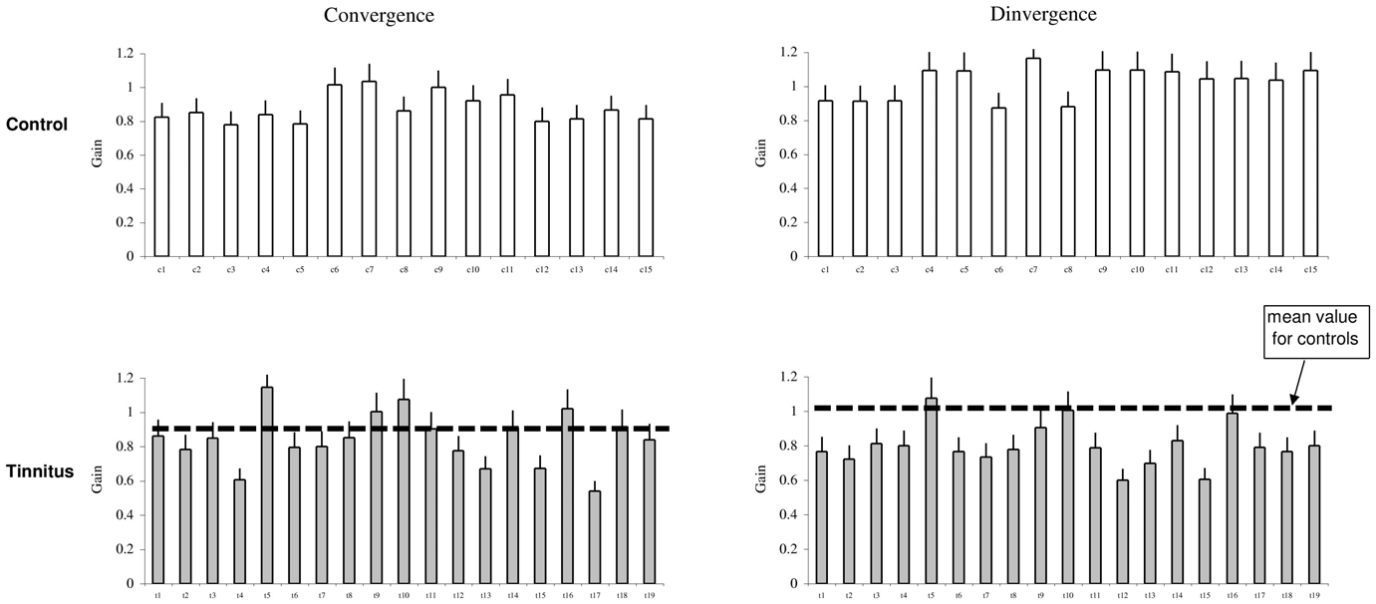
The accuracy of vergence eye movements. Individual mean of accuracy with standard deviation for convergence and divergence in control and tinnitus subjects.

Figure 4 shows the individual mean gain values of vergence with the standard deviation in control and tinnitus, respectively. ANOVA applied on the mean gain values showed a statistically significant effect of group (F_1,32_ = 11.54, p<0.01), i.e. the gain was lower in tinnitus than in controls, and a significant effect of type of vergence (F_1,32_ = 7.59, p<0.01), i.e. the gain is higher for divergence than for convergence. A significant interaction is found between group and vergence (F_1,32_ = 25.01, p<0.001). The effect of group is for divergence only (p<0.01).

### Peak velocity

**Figure 5:**
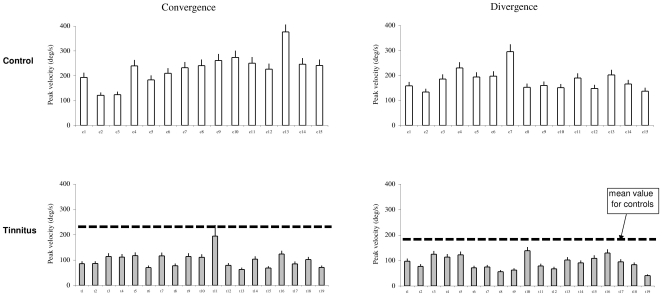
The peak velocity of vergence eye movements. Individual mean peak velocity with standard deviation for convergence and divergence in control and tinnitus subjects.

Figure 5 shows the individual mean values of peak velocity with the standard deviation for convergence and divergence in controls and tinnitus, respectively. ANOVA applied on the mean values of peak velocity shows statistically significant effect of the group (F_1,32_ = 58.75, p<0.001), i.e. lower peak velocity for tinnitus than for controls, and a significant effect of type of vergence (F_1,32_ = 7.10, p<0.05), i.e. higher peak velocity for convergence than for divergence. There is no effect of interaction between group and vergence (F_1,32_ = 1.34, p<0.26).

### Duration

**Figure 6:**
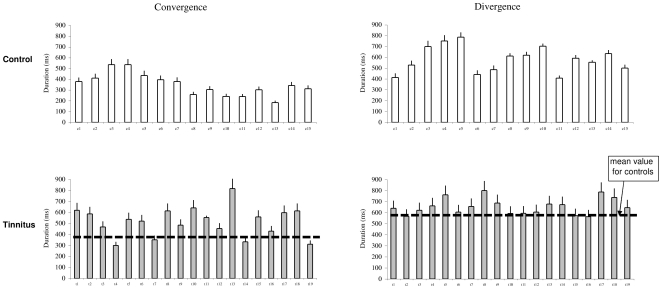
The duration of vergence eye movements. The Individual mean duration with standard deviation for convergence and divergence in control and tinnitus subjects.

Figure 6 shows the individual mean duration of vergence with the standard deviation in controls and tinnitus, respectively. ANOVA applied on the mean duration shows statistically significant effect of group (F_1,32_ = 9.92, p<0.01), i.e. longer duration for tinnitus than for controls, and effect of type of vergence (F_1,32_ = 47.17, p<0.001), i.e, longer duration for divergence than for convergence. There is no effect of interaction between group and vergence (F_1,32_ = 3.75, p>0.05).

**Figure 7:**
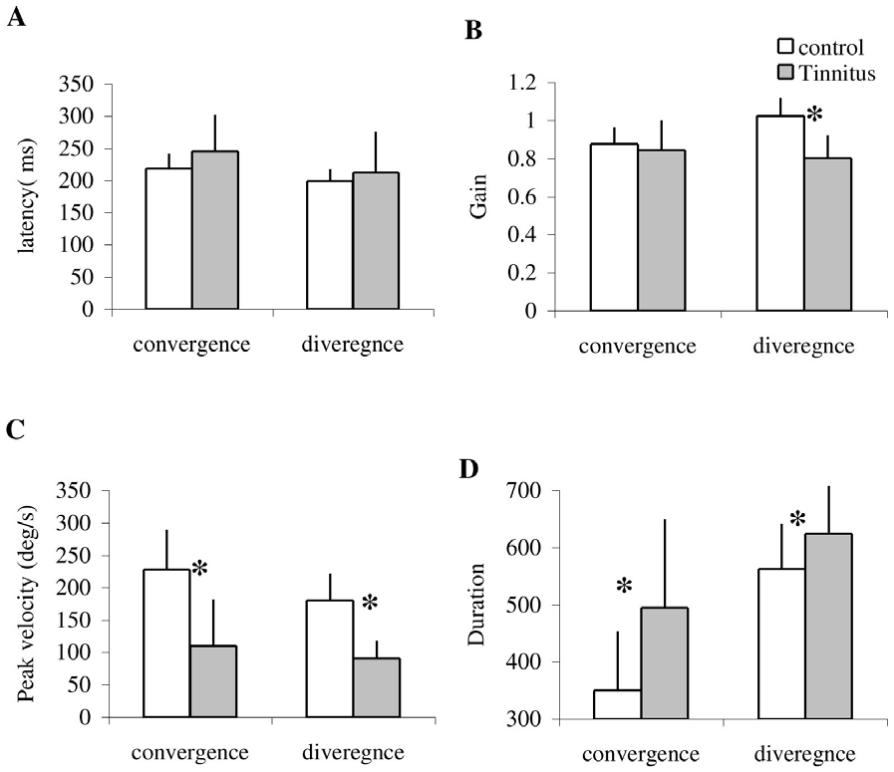
Group mean values of each parameter. Latency (A), Gain (B), Peak velocity (C) and Duration (D) for convergence and divergence in control and tinnitus subjects. Asterisks show statistically significant difference between controls and tinnitus.

Group mean values of all these parameters are summarized in Figure 7.

### Saccades during vergence

**Figure 8:**
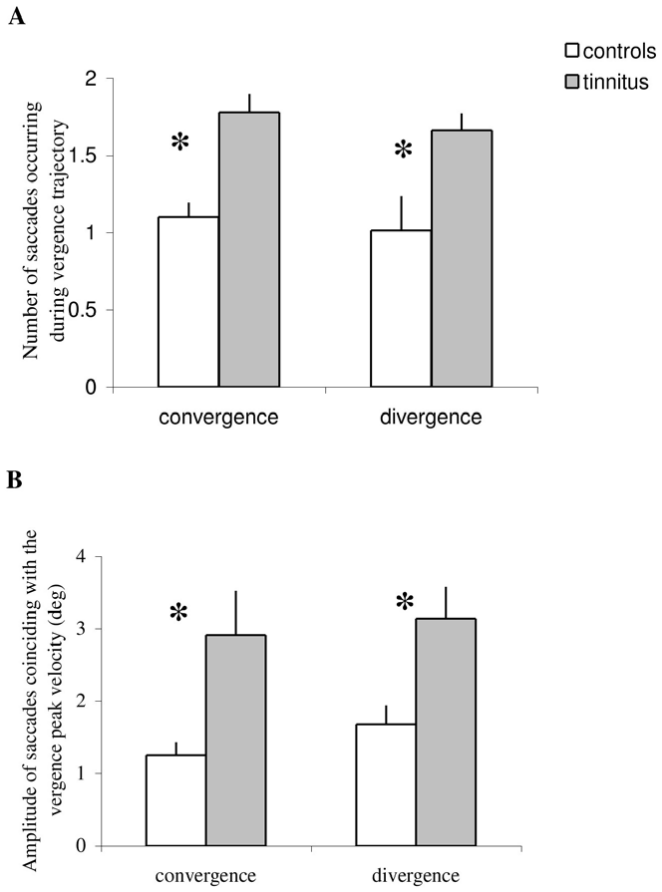
Embedded saccades. (A) Group mean number of embedded saccades during complete execution of the vergence–total trajectory seen example in Fig. 2 from ‘i’ to ‘e’. (B) the group mean amplitude of the subgroup of saccades coinciding with the peak velocity of vergence. Tinnitus subjects show significantly more embedded saccades, and higher amplitude of coinciding saccades than controls (asterisks).

Figure 8A presents the group mean number of embedded saccades during the total period of vergence execution; the group mean amplitude of the subgroup of saccades coinciding with the peak velocity of vergence is shown in Fig. 8B. ANOVA applied on the mean numbers shows statistically significant effect of the group (F_1,32_ = 16.43, p<0.001), i.e. more embedded saccades for tinnitus than for controls. ANOVA applied on the mean amplitude of such saccades coinciding with the peak velocity of vergence shows statistically significant effect of the group (F_1,32_ = 7.42, p<0.01), i.e. higher amplitude of coinciding saccades for tinnitus than for controls. These significant group effects occur for both convergence and divergence.

**Figure 9:**
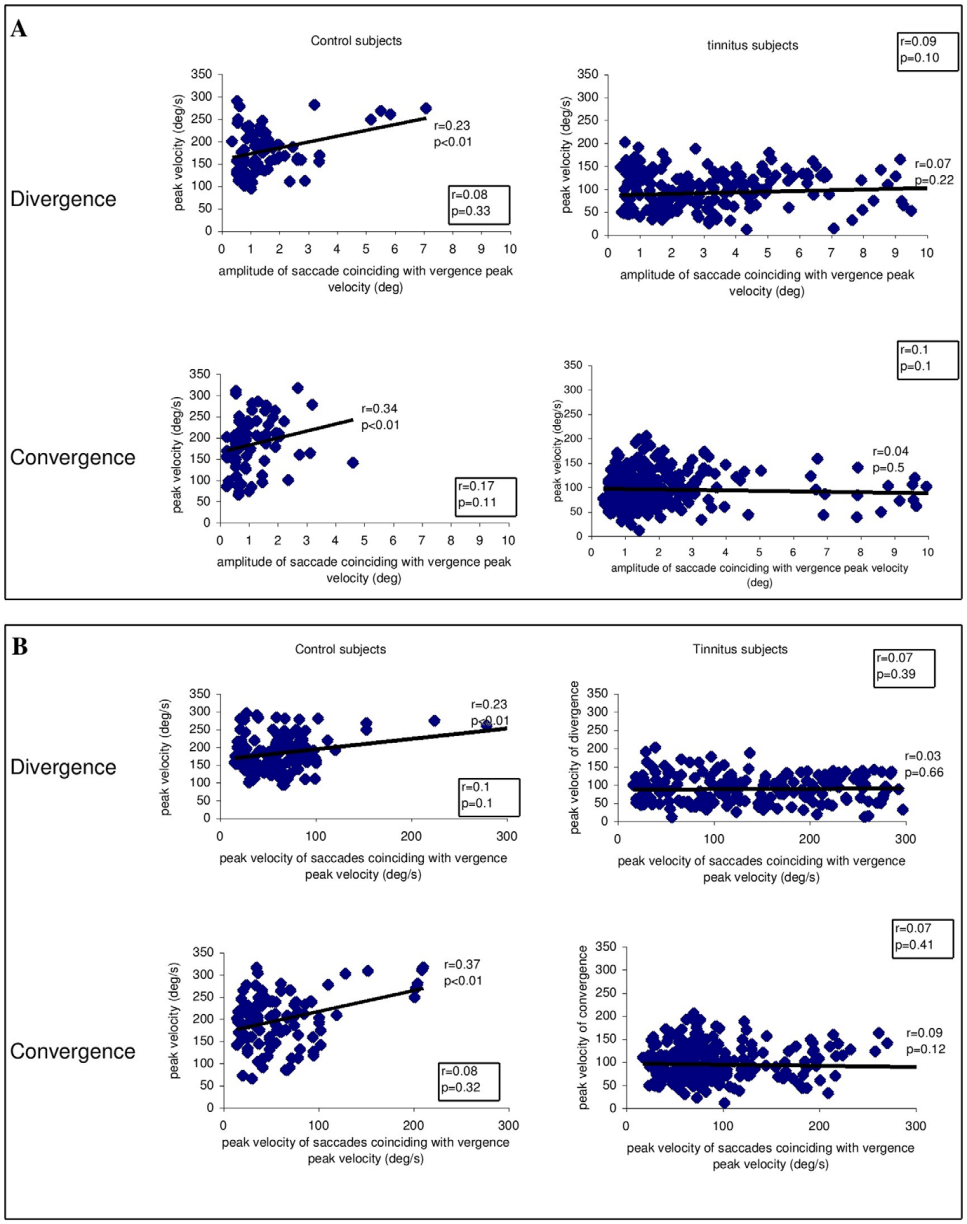
The correlation between the vergence and the coinciding saccades. (A) The correlation between the peak velocity of vergence and the amplitude of saccades coinciding with the peak velocity of vergence; (B) the correlation between the peak velocity of vergence and the peak velocity of saccades coinciding with the peak velocity of vergence. Correlation coefficients ‘r’ and levels of significance are shown next to each cluster. Positive significant correlations for both divergence and convergence occur for controls but for tinnitus patients. Values in boxes indicate correlations for the subgroup of saccades with amplitudes equal or less than 4°; no significant correlation exists for controls or tinnitus.

Figures 9A present the correlation between the peak velocity of vergence and the amplitude of the saccades coinciding with the peak velocity of vergence; the correlation between the peak velocity of vergence and the peak velocity of the coinciding saccades is shown in Figure 9B. The Spearman test shows significantly positive correlation for controls only: the higher the amplitude of the coinciding saccades, the higher the peak velocity of vergence. Also, the higher the peak velocity of the coinciding saccades, the higher the peak velocity of vergence. Yet, if we exclude a few saccades with larger amplitudes than 4°, the correlations are not significant anymore (see correlation coefficients and p values in box in each figure). For tinnitus subjects the peak velocity of vergence is not correlated with the amplitude or the peak velocity of coinciding saccades even though many of these saccades are of large amplitude. In summary, in controls if a large saccade (>4°) occurs during the peak velocity of vergence, vergence velocity increases, while for tinnitus patients no such increase occurs.

**Figure 10:**
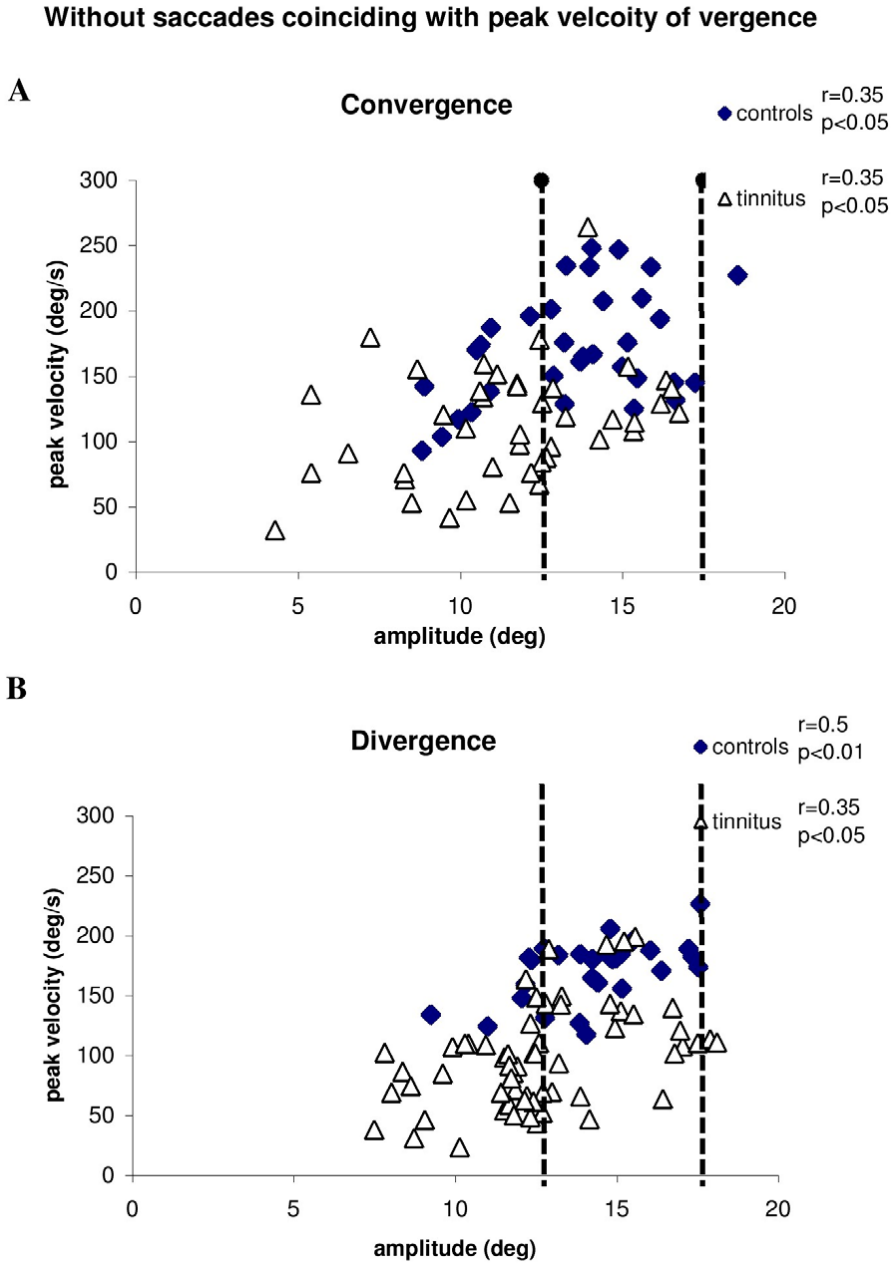
The main sequence of vergence. The correlation between the peak velocity and the amplitude of convergence (A) and of divergence (B) without coinciding saccades. All correlation coefficients and levels of significance are shown next to each cluster. Correlations are positive and significant, i.e. the peak velocity of vergence increases as the vergence amplitude increases for both controls and tinnitus subjects.

Figure 10 presents the correlation between the peak velocity and the amplitude of vergence without coinciding saccades. The Spearman test shows a significantly positive correlation for both controls and tinnitus subjects (all p<0.05), i.e. the peak velocity of vergence increases as the vergence amplitude increases. Yet, as it can be seen the peak velocity values from tinnitus subjects are, in general, lower than those from controls even for the range of amplitudes between 12.5 and 17.5 degrees (see dotted lines).

**Figure 11:**
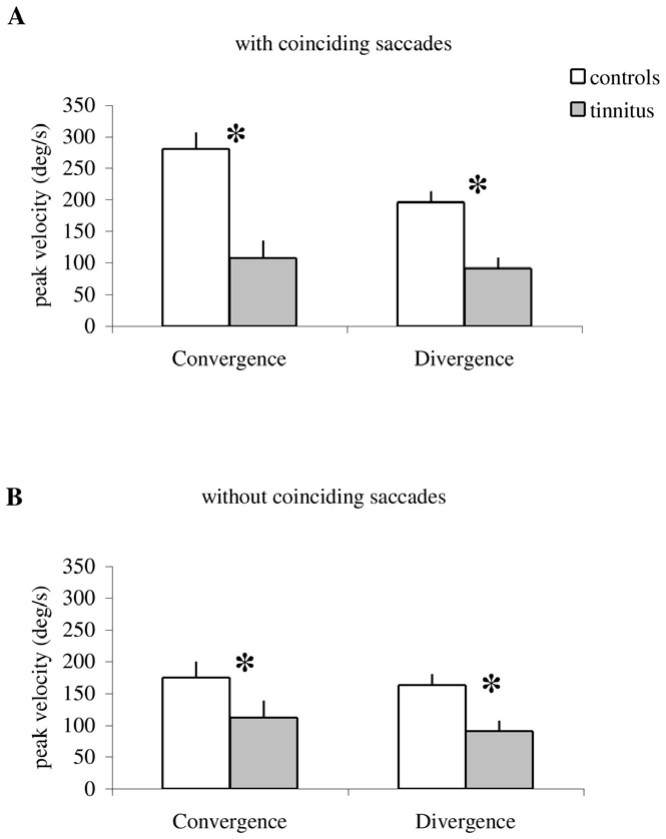
Group mean peak velocity of vergence. With saccades coinciding with the peak velocity of vergence (A) and without such saccades (B). Tinnitus subjects show lower peak velocity of vergence than controls for all cases (asterisks). Also, for controls only, vergence velocity is higher with coinciding saccades than without such saccades.

Figure 11 summarizes group mean peak velocity of vergence with coinciding saccades (A) and vergence without coinciding saccades (B) for controls and tinnitus. ANOVA applied on the group mean peak velocity of vergence shows statistically significant effect of the group for both cases with (F_1,32_ = 41.82, p<0.001) and without (F_1,32_ = 59.41, p<0.001) coinciding saccades, i.e. the peak velocity of vergence is lower in tinnitus than in controls for both convergence and divergence. In addition, for controls the peak velocity is higher for vergence with coinciding saccades than for that without coinciding saccades ((F_1,32_ = 39.02, p<0.001). This is a case for both convergence and divergence. Yet, for tinnitus there is no difference of the peak velocity between these two cases (F_1,32_ = 0.001, p = 0.92). Thus, the peak velocity of vergence is always lower for tinnitus, with or without coinciding saccades.

## Discussion

This study showed no latency abnormalities but low accuracy for divergence, low peak velocity and long duration of both convergence and divergence, more frequent embedded saccades and of higher amplitude of coinciding saccades in tinnitus relative to controls. Tinnitus subjects did not show normal saccade-vergence interaction, that is, saccades did contribute to increase of the velocity of vergence as seen in controls.

### Convergence–divergence difference

Before discussing the group effects we will discuss the different properties of convergence versus divergence confirmed for both groups (controls and tinnitus). Note that shorter latency for divergence than for convergence is the case for both controls and tinnitus. This result is compatible with our previous study (Yang & Kapoula 2004; Yang et al. 2009) and other studies (Alvarez et al. 2002, 2005). Such difference could be explained by Patel's model (a neural network model): two types of disparity encoders (disparity-tuned cells) employed in this model, convergence and divergence encoders. In other words, the divergence is not simply a negative convergence movement (i.e. passive relaxation of convergence) but a separate neurophysiological system. The neuro-control strategy of these two systems may be different (Alvarez et al. 2005). Perhaps this model can also explain the different dynamics between convergence and divergence (shorter duration and higher peak velocity for convergence than for divergence).

### Substrate of vergence

Different cortical-subcortical-cerebellar areas are known to be involved in the generation of vergence [22]. Triggering of vergence is believed to involve cortical areas, such as posterior parietal cortex and frontal eye field [9]. Dynamics of vergence is also related the function of cortical-brainstem-cerebellar circuits. For instance, Gamlin & Yoon [8] identified some neurons that displayed phasic activity correlated with vergence dynamics and some neurons that displayed tonic activity correlated with vergence angle and other neurons that displayed both phasic and tonic activity. Judge and Cumming [12] also reported 19 neurons in midbrain discharged in relation to the vergence response. As mentioned, Nitta et al. [17] found that the majority of vergence related P-cells in the cerebellum carried convergence signals with both eye velocity and position sensitivities, and they discharged before the onset of convergence eye movements. Muscimol infusion into the sites where convergence P-cells were recorded resulted in a reduction of peak convergence eye velocity. The possible pathophysiological vergence mechanisms will be discussed below.

### Abnormalities of vergence dynamics in tinnitus-Mechanisms

For many years, it has been advocated that ST could be solely related to an otogenic peripheral dysfunction within ear structures (external, middle, inner ear) [30]. Recently, research stressed the implication of central involvement in ST percept and in ST related distress leading to the conclusion that in most cases the real culprit is in the brain. Importantly, researchers found that completely dubbing the auditory nerve, which passes sound information from the ear to the brain, failed to stop the ringing of tinnitus in some patients. This shows that the sounds can originate in the brain. For example, for the first time, Arnold et al. [31] using positron emission tomography (PET) in nine tinnitus patients and showed significantly increased metabolic activity in the left or right primary auditory cortex (Pac, Brodmann area 41). Giraud et al. [32] used PET and demonstrated that phantom auditory sensation increased regional cerebral blood flow bilaterally in temporo-parietal association auditory areas but not in the primary auditory cortex in patients with tinnitus. Recently, Lockwood et al. [33]found that in patients with gaze-evoked tinnitus (GET), tinnitus loudness and pitch was seen in the auditory lateral pontine tegmentum or auditory cortex; moreover, GET associated eye movements activated the cuneus and cerebellar vermis. Muhlau et al. [34] used high-resolution magnetic resonance imaging (MRI) and found gray-matter increase only at the thalamic level in tinnitus sufferers.

All areas involved in tinnitus mentioned above, particularly cerebral vermis and related brainstem areas, are also involved in the control of vergence. For example, neurons in flocculus of cerebellum discharge according to the angle of vergence [23]; the nucleus reticularis tegmenti pontis, which projects to the cerebellum, and the posterior nucleus interpositus (the globuse and emboliform nuclei in humans) contain cells that also discharge in relation to different aspects of the near response including vergence [16]. In humans, problems of vergence are reported in patients with progressive superanuclear palsy or Parkinson's disease. In addition, numerous studies reported that saccades can increase the speed of vergence [35], [36], [37]. Such benefit for the vergence from saccades could be interpreted by the multiply model [37] according to which speeding of vergence by the saccade may be the result of the inhibition of a subgroup of vergence-related neurons by the saccadic omnipause neurons (OPNs in the nucleus raphe interpositus). Also by an alternative model [35], [36] according to which that vergence enhancement is the result of a multiplicative interaction between a weighted saccadic burst signal and the vergence motor error driving the vergence system. An internal mechanism of feedback would control the movement progression; this feedback is suggested to be cortical-midbrain-cortical loop. Our results show that tinnitus patients cannot increase their vergence velocity even when vergence is accompanied by saccades of large amplitude. These results suggested that there are also problems for the central system controlling the saccade-vergence interaction, OPNs or the cortical-midbrain-cortical loop. Thus we suggest that the degree of deficit of vergence could be an indicator of deficit of the cortical-brainstem-cerebellar circuit.

Eye movements are excellent tools for investigating brain function as they can provide information about function at multiple levels. The observation here for vergence together with our previous studies in smooth pursuit and optokinetic nystagmus [4], and other studies in saccades [5] for tinnitus showing abnormalities for almost types of eye movements are compatible with the idea of dysfunction of structures involved in many types of eye movements (such as the cerebellum) and mild dysfunction. As mentioned in the Introduction section, the cerebellum is involved both for adaptive control and on-line control of vergence [16], [17], [18], [19], [20]. Future studies testing the capacity of tinnitus patients to adapt to prisms are of interest. As such capacity depends on the cerebellum we predict limitations for such tinnitus patients. The oculomotor findings reported here are in line with observations of functional magnetic resonance imaging [33] describing cerebellar activity in patients with GET. Perhaps tinnitus and vergence dysfunction are both manifestations of mild cerebellar syndrome including abnormality of cross-modality interactions between vergence and auditory signals.

Given that saccades and vergence are both gifted by plasticity and adaptive capacity, eye movement training may be used to reduce vergence abnormality. In a retrospective analysis in patients with acquired brain injury Ciuffreda et al. [26] observed lasting vergence abnormalities. Ciuffreda et al. [38] showed that vision therapy for oculomotor dysfunction can be efficient as considerable residual neural plasticity exist. Therefore, vergence orthoptic training could be useful alleviating visual symptoms and perhaps abnormal oculomotor-auditory interactions. Thus usefulness of oculomotor reeducation in patients with somatic tinnitus could be interestingly more widely tested in future clinical research.

Finally, vergence abnormalities reported here could be present only for patients who can modulate their tinnitus. It will be interesting to study patients with tinnitus without movement modulation. Future research is then needed to determine if the vergence problem is due to ST itself or is related to the specific ability of these patients to modulate their tinnitus by movements.
